# Varicella Zoster Virus Reactivation Following COVID-19 Vaccination: A Systematic Review of Case Reports

**DOI:** 10.3390/vaccines9091013

**Published:** 2021-09-11

**Authors:** Konstantinos Katsikas Triantafyllidis, Panagiotis Giannos, Imran Tariq Mian, George Kyrtsonis, Konstantinos S. Kechagias

**Affiliations:** 1 Department of Nutrition & Dietetics, Musgrove Park Hospital, Taunton & Somerset NHS Foundation Trust, Taunton TA1 5DA, UK; k.katsikas-triantafyllidis18@alumni.imperial.ac.uk; 2 Society of Meta-Research and Biomedical Innovation, London W12 0BZ, UK; panagiotis.giannos19@imperial.ac.uk; 3 Department of Life Sciences, Faculty of Natural Sciences, Imperial College London, London SW7 2AZ, UK; 4 Brighton and Sussex Medical School, Falmer BN1 9PX, UK; i.mian1@uni.bsms.ac.uk; 5 Department of General Surgery, Croydon University Hospital, Croydon, London CR7 7YE, UK; georgios.kyrtsonis@nhs.net; 6 Department of Metabolism, Digestion and Reproduction, Faculty of Medicine, Imperial College London, London W12 0NN, UK

**Keywords:** coronavirus, SARS-CoV-2, COVID-19 vaccine, Varicella zoster virus, herpes zoster, systematic review, case reports, case series

## Abstract

The newly developed COVID-19 vaccines have established a safe profile, yet some individuals experience a wide range of adverse events. Recently, reactivation of varicella zoster virus (VZV) has been observed after administration of different COVID-19 vaccines, although causality remains a matter of debate. The aim of this systematic review was to examine the available literature and provide an overview of reported cases of VZV reactivation following COVID-19 vaccination. We identified 12 eligible articles which included 91 patients with herpes zoster (HZ) following COVID-19 vaccination. Hypertension was the main comorbidity present in 18% of patients (16/91). Additionally, 13% of patients (12/91) had an autoimmune condition with rheumatoid arthritis being the most common (4/12). Moreover, 10% of patients (9/91) were receiving immunosuppressants. The dermatomal distribution of skin lesions varied among patients, with the mammary region being most affected. On average, symptoms developed 5.8 days post-vaccination irrespective of dose and treatment with oral valacyclovir as a monotherapy was employed in most patients (23/91). HZ is possibly a condition clinicians may expect to encounter in patients receiving COVID-19 vaccines. While causality has not yet been established increased awareness and early recognition of the disorder would be crucial for the optimal management of these patients.

## 1. Introduction

In March 2020, the World Health Organization (WHO) declared a public health emergency of international concern towards an atypical viral pneumonia outbreak first described in Wuhan, Hubei Province of China. The cause was a novel coronavirus strain termed, Severe Acute Respiratory Syndrome Coronavirus 2 (SARS-CoV-2) [1,2]. Since then, the coronavirus disease (COVID-19) has spread to all countries across the globe and hundreds of millions of people have been affected, making the imperativeness for developing a safe and effective vaccine, vital to controlling the pandemic and its socioeconomic implications [3].

To date, the European Medicines Agency (EMA) has authorised four vaccines for use against COVID-19: COMIRNATY (the COVID-19 mRNA vaccine BNT162b2 by BioNTech–Pfizer); COVID-19 Vaccine Moderna (mRNA-1273 by Moderna); VAXZEVRIA (ChAdOx1-nCoV19 by AstraZeneca-Oxford University); and COVID-19 Vaccine Janssen (Ad26.COV2.S by Janssen) [4]. Roughly 4 billion vaccine doses have been administered worldwide with overall effectiveness against severe infection varying from 70–95% [5,6,7,8].

Contrary to vaccines such that by AstraZeneca-Oxford University and Janssen, which employ an adenovirus to deliver the viral genome for the expression of the SARS-CoV-2 spike (S) protein, the mRNA vaccines of Moderna and BioNTech–Pfizer provides a novel sequence-optimised mRNA that relies on lipid nanoparticles for delivery [9]. Although the mechanism of action among all four vaccines is different, they share numerous commonly reported adverse events including, pain at the injection site, pyrexia, headache, nausea and vomiting, all of which can develop after the first and/or second dose [10,11,12]. Less frequently observed adverse reactions include dermatological complications, such as lime maculopapular eruptions, morbilliform rashes, urticaria, chickenpox-like lesions and the more recently reported reactivation of varicella zoster virus (VZV) [13,14].

VZV is a human neurotropic herpes virus that establishes in ganglionic neurons and causes varicella (chickenpox) [15]. Usually, VZV is presented in the form of herpes zoster (HZ), which is clinically characterised by a painful, unilateral vesicular eruption in the dermatome innervated by the ganglion [15]. VZV reactivation is influenced by the immune status and age of the patients, with altered immunocompromised state and ageing being major risk factors [16]. To date, vaccine administration has not been considered as a triggering factor.

The aim of this systematic review was to comprehensively examine the currently available literature and provide an overview of the reported cases of VZV reactivation following vaccination against COVID-19.

## 2. Methods

This review was reported based on the “Preferred Reporting Items for Systematic Reviews and Meta-Analyses” (PRISMA) guidelines [17].

### 2.1. Literature Search

Two reviewers searched PubMed and Scopus databases from inception until July 2021. The search included the following terms: “(COVID 19 vaccin*) OR (COVID-19 vaccin*) OR (SARS-COV2 vaccin*) OR (SARS-COV-2 vaccin*) AND (herpes zoster) OR (varicella zoster) OR (Shingles)”. No restrictions regarding study design, geographic region, or language were applied. A manual search of references cited in the selected articles and published reviews was also used for undetected studies. Discrepancies in the literature search process were resolved by a third investigator.

### 2.2. Eligibility Criteria

We included studies that provided data for VZV reactivation cases following COVID-19 vaccination with at least one dose. All study designs were considered eligible for inclusion. Review articles, abstracts submitted in conferences and non-peer reviewed sources were not eligible for inclusion. Studies on in vitro and animal models were excluded.

### 2.3. Data Extraction and Handling

In all studies, patient data was retrieved and handled by two authors who conducted the data extraction independently. We collected the following information: sex, age, comorbidities, type of vaccine administered, number of doses received, days of HZ onset after vaccination, involved dermatome, HZ treatment, duration of HZ treatment and prior history of VZV infection and vaccination. Any disagreements were discussed and resolved by a third author.

### 2.4. Quality Assessment

The studies were evaluated using the criteria established by the Task Force for Reporting Adverse Events of the International Society for Pharmacoepidemiology (ISPE) and the International Society of Pharmacovigilance (ISoP) [18]. The assessment was based on the adequate reporting of 12 different elements namely: title, patient demographics, current health status, medical history, physical examination, patient disposition, drug identification, dosage, administration/drug reaction interface, concomitant therapies, adverse events, and discussion. The studies scored either 0 (absence of information) or 1 (containing the information) for every element.

## 3. Results

### 3.1. Study Characteristics

**Figure 1:**
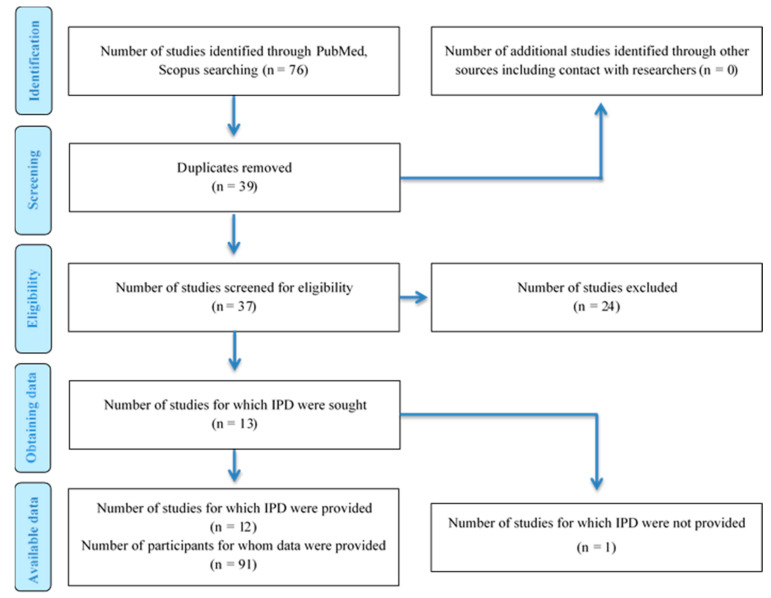
Prisma flowchart. IPD: Individual Patient Data.

The initial literature search yielded 76 publications. In the first screening, 24 studies were excluded as irrelevant. Additionally, one study did not provide individual data and was therefore, excluded. After this exclusion, 12 studies were eligible for the systematic review (Figure 1). Seven of the studies were case reports, four were case series and one was cross-sectional. Six of the studies were conducted in Asia, five studies in Europe and one in America.

We identified a total of 91 cases of VZV reactivation following vaccination against COVID-19. Forty-one participants were males and 50 were females with a mean age of 62 years. Hypertension was the main comorbidity present in 18% of the patients (16/91) followed by dyslipidaemia in 5% of patients (5/91). Additionally, 13% of the patients (12/91) had an autoimmune condition with rheumatoid arthritis being the most common (4/12). Moreover, 10% of the patients (9/91) were receiving an immunosuppressive medication. Prior history of VZV infection and prior vaccination against VZV was reported in 15% (14/91) and 13% (12/91) of the patients, respectively. Of these, seven patients did not report previous VZV reactivation, and 11 patients were not vaccinated against VZV. Finally, 8% of the patients (7/91) had previous history of COVID-19 infection.

The majority of the patients (37/91) received the COMIRNATY, followed by COVID-19 Vaccine Moderna (25/91), while only a small fraction of participants (12/91) received VAXZEVRIA. Three case studies did not mention the vaccine type administered (Table 1).

The bigger proportion of patients developed symptoms after the first dose (53/91), while the rest (35/91) after the second dose. Two studies did not report the number of doses that the patients received before developing symptoms. On average, the symptoms developed 5.8 days after the administration of the vaccine irrespective of the dose. The main reported location was the mammary region (9/91) followed by the back (8/91). The most commonly involved dermatome was T4 (12/91) (Table 1 and Table 2).

Most cases (23/91) were treated with valacyclovir as monotherapy while a fraction of the patients (12/91) received valacyclovir in combination with a second antiviral agent (i.e., Fusidic acid, Acyclovir). The treatment course was reported in 50% of the studies (6/12) and varied from 10 days to 6 weeks with the most common treatment period being 1 week (5/6) (Table 1).

### 3.2. Quality of the Studies

The mean quality score indicated that the studies reported on average 10 of the recommended 12 elements, defined by the guidelines. Only five studies had a perfect score of 12 while the second most common score was 10. The most frequently missing information were the following: patient disposition (4/12), drug identification (or in our case vaccine identification) (3/12) and adverse events after vaccine administration (4/12) (Table 3).

## 4. Discussion

In the current systematic review, we examined the potential association between COVID-19 vaccination and HZ reactivation. Our study included 12 reports which comprised of 91 patients in which HZ reactivation was reported after administration of different COVID-19 vaccines. Our findings revealed that dermatomal distribution of skin lesions was scattered among patients with HZ, but the mammary region was the most commonly affected anatomical site. The onset of lesions and symptoms started after administration of the first dose in the majority of cases and treatment with oral valacyclovir as a monotherapy was employed for most patients. The majority of the patients were older than 60 years of age and more than a fifth of patients suffered from an autoimmune disorder and/or were receiving immunosuppressants.

VZV is a pathogenic human alpha-herpes virus that causes varicella (chickenpox) as a primary infection, which usually occurs in children [27]. Following primary infection, this neurotropic virus becomes latent in neurons of dorsal root ganglia, cranial nerve ganglia, and autonomic ganglia [30]. During latency, VZV genome is transcriptionally silent in a circular single episomal unit [31]. Although the molecular events by which the VZV genome is silenced in the neuronal nucleus are ill-defined, different mechanisms have been proposed including the nuclear domain-10-induced silencing of VZV gene expression [27].

Up to decades later, reactivation of latent VZV may arise and cause HZ, which typically presents as painful or pruritic cutaneous vesicular eruptions that follow a characteristic dermatomal distribution [27]. Viral reactivation may arise spontaneously or following a plethora of triggering factors. Of particular interest, viral reactivation appears more frequently in older individuals because of their diminished cell-mediated immunity, a phenomenon known as immunosenescence [32]. Other stimuli include immunosuppression from disease or drugs, trauma, X-ray irradiation, infection, and malignancy [16,33]. In line with the above, most of the reported patients in our review were of older age with a percentage suffering from autoimmune disorders and/or receiving immunosuppressant agents.

The newly developed COVID-19 vaccines have established a safe profile. However, some individuals still experience a wide range of mild to moderate side effects [10,11,12]. While reactivation of VZV was not reported as a side effect in any of the initial vaccination trials [10,11,12], a total of 2527 cases of HZ infection were published as Yellow Card reports by the Medicines and Healthcare products Regulatory Agency (MHR) in the United Kingdom [34]. Specifically, 22 cases were linked to COVID-19 Vaccine Moderna, 1443 to VAXZEVRIA, 1062 to COMIRNATY, while no data were reported for COVID-19 Vaccine Janssen [34]. Interestingly, co-occurrence of HZ and other vaccines, including influenza, hepatitis A, and rabies, has been also reported, although these have been considered extremely rare [35].

In patients with severe disease, COVID-19 produces an immunosuppressive state that is described by a quantitative decrease in T lymphocytes, particularly those of CD4+ T cells, CD8+ T cells, and natural killer cells [36]. It has been hypothesized that these changes in immune status including lymphopenia and lymphocyte exhaustion may potentially lead to HZ reactivation. Indeed, 27 cases of HZ following COVID-19 have been identified and most frequently occurred within 2 weeks upon infection [37].

The incidence of HZ reactivation following COVID-19 vaccination appears contradictory. Stimulation of the immune system following vaccination induces a strong T-cell response which often persists. Particularly, a cellular response with increased CD8+ T cell and T helper type 1 CD4+ T cells has been clearly documented shortly after booster doses for Pfizer and Moderna [38]. A compelling hypothesis for this phenomenological paradox has emerged and suggests that VZV-specific CD8+ cells are not, temporarily, capable of controlling VZV after the massive shift of naïve CD8+ cells in the setting of SARS-COV-2 vaccination [22].

Other possible explanations have also been proposed and focus on toll-like receptors (TLR) signalling, which is often implicated in the reactivation process of herpes viruses as a maintenance mechanism in the host [39,40]. Particularly, abrogations in TLR expression among vaccinated individuals have been linked with marked induction of type I interferon (IFN) and potentiation of pro-inflammatory cytokines, which although promote T cell immunity and initiate an antibody-secreting memory B cell response, may negatively modulate antigen expression while potentially contributing to HZ reactivation [25].

### Strengths and Limitations

To the best of our knowledge, our study is the first to review the association between COVID-19 vaccination and VZV reactivation. Our findings present a comprehensive overview of the currently available literature and highlight published data with rigorous quality assessment of included studies.

However, our study is not without any limitations. A broader drawback underlies the low-quality nature of case reports and case series included in our review, which hinders the validity and scope of conclusions that can be reached. In fact, the potential risk of bias of these studies is inevitable, as these are especially vulnerable to the risk of overinterpretation and selection bias. In this way, their reported data although intriguing may be far from the truth without reflecting a valid description. Thus, causality cannot be inferred and requires insight from data that compare HZ reactivation in vaccinated and non-vaccinated individuals.

## 5. Conclusions

Although the currently available COVID-19 vaccines have established a safe profile, patients still experience mild to moderate side effects including dermatological complications. Herpes zoster is possibly a condition physicians and other healthcare professionals may expect to see in patients receiving COVID-19 vaccines. While the above mild adverse event is still underreported and causality is not yet confirmed, the increased awareness of clinicians and the early recognition of the disorder is important for the optimal management of these patients. Prophylaxis with oral valacyclovir for high-risk individuals may also be considered.

## Figures and Tables

**Table 1:** Characteristics of the included studies.

Author, Year	Country	Study Design	N of Patients (Males/Females)	Mean Age	Comorbidities (N of Cases)	Vaccine Type (N of Patients)	Vaccine Doses before Symptoms (N of Patients)	Days of HZ Onset after Vaccination (Mean)	Treatment (Duration)
Aksu, 2021 [19]	Turkey	Case study	1 (1/0)	68	Hypertension (1) Dysrhythmia (1) Anxiety (1)	N/A	2nd dose (1)	5	Valaciclovir (7 days)
Alpalhão, 2021 [20]	Portugal	Case series	4 (1/3)	69	Hallux valgus (1) Systematic lupus erythematosus (1) APS (1) Plaque-type psoriasis (1) Psoriatic arthritis (1) Hemophilia A (1) Hypertension (1)	Pfizer-BNT162b2 (2) AstraZeneca- ChAdOx1-nCoV19 (2)	1st dose (2) 2nd dose (2)	4	Valacyclovir (N/A)
Arora, 2021 [21]	India	Case study	1 (1/0)	60	Hypertension (1) Diabetes Mellitus (1)	AstraZeneca- ChAdOx1-nCoV19 (1)	1st dose (1)	4	Valacyclovir, Fusidic acid (7 days)
Bostan, 2021 [14]	Turkey	Case study	1 (1/0)	78	CAD (1) CVA (1) Hypertension (1) COPD (1)	N/A	N/A	5	Valacyclovir (7 days)
Català, 2021 [22]	Spain	Cross sectional	41 (16/25)	61	Atopic dermatitis (1) Allergic Asthma (1) Allergic Rhinitis (2) Urticaria (2)	Pfizer-BNT162b2 (28) Moderna-mRNA-1273 (6) AstraZeneca- ChAdOx1-nCoV19 (7)	1st dose (26) 2nd dose (15)	6.9	N/A
Chiu, 2021 [23]	Taiwan	Case series	3 (3/0)	53	N/A	Moderna-mRNA-1273 (1) AstraZeneca- ChAdOx1-nCoV19 (2)	1st dose (3)	3.6	Acyclovir (7 days)
Eid, 2021 [24]	Lebanon	Case study	1 (1/0)	79	Hypertension (1) CAD (1) ANCA-related glomerulonephritis (1)	N/A	N/A	5	N/A
Furer, 2021 [25]	Israel	Case series	6 (0/6)	49	Sjogren’s syndrome (1) RA (4) ILD (1) UCTD (1) APS (1)	Pfizer-BNT162b2 (6)	1st dose (1) 2nd dose (5)	8	Valacyclovir, Acyclovir (7 days)
Lee, 2021 [26]	USA	Case series	20 (10/10)	56	Crohn’s disease (1) Hypertension (7) Polycythaemia Vera (1) Dyslipidaemia (2) Osteopenia (1) Diabetes Mellitus (1) CKD (1) Anaemia (1) Gout (2) Heart failure (1) Atrial Fibrillation (1) Hypothyroidism (1)	Moderna-mRNA-1273 (14) Pfizer-BNT162b2 (6)	1st dose (15) 2nd dose (5)	6.9	Valacyclovir (N/A)
Psichogiou, 2021 [27]	Greece	Case series	7 (4/3)	77	Osteoporosis (1) Dyslipidaemia (2) Prostate cancer (1) Hypertension (3) Hyperuricemia (1) COPD (1) Heart failure (1) CKD (1)	Pfizer-BNT162b2 (7)	1st dose (1) 2nd dose (6)	9	Valacyclovir (N/A)
Rodriguez-Jimenez, 2021 [28]	Spain	Case series	5 (2/3)	48	Hypertension (1)	Pfizer-BNT162b2 (5)	1st dose (3) 2nd dose (2)	5.4	N/A
Tessas, 2021 [29]	Finland	Case study	1 (1/0)	44	Dyslipidaemia (1)	Pfizer-BNT162b2 (1)	1st dose (1)	7	Valacyclovir (14 days)

ANCA: Antineutrophil Cytoplasmic Antibodies, APS: Anti-Phospholipid Syndrome, CAD: Coronary Artery Disease, CKD: Chronic Kidney Disease, COPD: Chronic Obstructive Pulmonary Disease, HZ: Herpes Zoster, LMX: Lidocaine, N/A: not available, RA: Rheumatoid Arthritis, UCTD: Undifferentiated Connective Tissue Disease.

**Table 2:** Dermatome and anatomical site involvement in the reported HZ cases.

Author, Year	Case Number	Dermatome/Anatomical Site
Aksu, 2021	Case 1	T3-T5 (Right chest)
Alpalhão, 2021	Case 1	5th cranial nerve
	Case 2	5th cranial nerve
	Case 3	C8
	Case 4	5th cranial nerve
Arora, 2021	Case 1	L2–L3 (Right leg)
Bostan, 2021	Case 1	T3–T5 (Right chest)
Català, 2021	Case 1–41	N/A
Chiu, 2021	Case 1	T8
	Case 2	T10
	Case 3	T11
Eid, 2021	Case 1	Right leg
Furer, 2021	Case 1	L5
	Case 2	5th cranial nerve
	Case 3	L1–L2
	Case 4	T10
	Case 5	T4
	Case 6	T6
Lee, 2021	Case 1	Mid-abdomen, right flank, and right mid-back
	Case 2	Left axilla, left shoulder, left triceps
	Case 3	Right chest
	Case 4	Left back, left shoulder, left triceps
	Case 5	Right neck, right collarbone, right lower jaw
	Case 6	Left axilla, left upper chest
	Case 7	N/A
	Case 8	T1
	Case 9	Right back, right flank
	Case 10	Mid chest, right arm
	Case 11	Left flank
	Case 12	Left axilla, left triceps, left scapula
	Case 13	Right flank
	Case 14	Right forehead
	Case 15	Right flank, back
	Case 16	Under right eye
	Case 17	Left eyebrow
	Case 18	Left back, left abdomen
	Case 19	Right back, right arm
	Case 20	Left arm, left upper back, left breast
Psichogiou, 2021	Case 1	Lumbar region
	Case 2	Thoracic region (Right chest)
	Case 3	5th cranial nerve
	Case 4	Thoracic region (Right chest)
	Case 5	Thoracic region (Right chest)
	Case 6	5th cranial nerve
	Case 7	Thoracic region (Right chest)
Rodriguez, 2021	Case 1	C6
	Case 2	Dorsal 2-Dorsal 4
	Case 3	Dorsal 4
	Case 4	5th cranial nerve
	Case 5	Dorsal 5
Tessas, 2021	Case 1	C5-C6 (Left upper back, left arm)

HZ: Herpes Zoster, N/A: not available.

**Table 3:** Quality assessment of the included studies.

Author, Year	Title	Demographics	Current Health Status	Medical History	Physical Examination	Patient Disposition	Drug Identification	Dosage	Administration Drug- Reaction Interface	Concomitant Therapies	Adverse Events	Discussion	Overall Rating
Aksu, 2021	●	●	●	●	●	●	○	●	●	●	○	●	10
Alpalhão, 2021	●	●	●	●	●	●	●	●	●	●	●	●	12
Arora, 2021	●	●	●	●	●	●	●	●	●	●	●	●	12
Bostan, 2021	●	●	●	●	●	○	○	●	○	●	○	●	8
Català, 2021	●	●	○	●	○	●	●	●	○	○	●	●	8
Chiu, 2021	●	●	●	●	●	●	●	●	●	○	○	●	10
Eid, 2021	●	●	●	●	●	○	○	●	○	●	●	●	9
Furer, 2021	●	●	●	●	●	●	●	●	●	●	●	●	12
Lee, 2021	●	●	●	●	●	○	●	●	●	○	●	●	10
Psichogiou, 2021	●	●	●	●	●	●	●	●	●	●	●	●	12
Rodriguez-Jimenez, 2021	●	●	●	●	●	○	●	●	●	●	○	●	10
Tessas, 2021	●	●	●	●	●	●	●	●	●	●	●	●	12

Key: ○ = 0, ● = 1.

## Data Availability

The data used to support the findings of this study are included within the article.
